# HistoMap: Reconstructing Spatially Resolved Single-Cell Profiles from Bulk RNA-Seq to Decipher the Immune-Excluded Microenvironment in Colon Cancer

**DOI:** 10.3390/ijms27125259

**Published:** 2026-06-10

**Authors:** Jia He, Yong Cao, Yan Liu, Xuan Zhang, Jianxin Ji, Hesong Wang, Yongzhen Song, Qiuju Zhang, Lei Cao

**Affiliations:** Department of Biostatistics, School of Public Health, Harbin Medical University, Harbin 150081, China

**Keywords:** single-cell reconstruction, histology–transcriptomic integration, colon cancer, tumor microenvironment, SPP1+ macrophages, immune-excluded phenotype

## Abstract

Bulk RNA-sequencing (bulk RNA-seq) averages gene expression across cell mixtures, obscuring single-cell heterogeneity and spatial architectures essential for understanding pathological processes. We developed HistoMap, a deep learning-based framework for single-cell spatial deconvolution. The model employs a two-stage pipeline: first, reconstructing high-fidelity single-cell profiles from bulk data using a β-variational autoencoder, and second, utilizing a Histological Vision Transformer (H-ViT) to map these cells to tissue coordinates via dual guidance from transcriptomic references and H&E-stained morphological constraints. HistoMap demonstrated superior performance across diverse human tissues, achieving a Pearson Correlation Coefficient (PCC) of 0.800 on external validation. Application to 14 colorectal cancer cases revealed a Macro_SPP1-mediated desmoplastic barrier. SPP1+ macrophages act as spatial hubs at the invasive front, forming a physical “sequestration belt” that functionally excludes cytotoxic T cells from the tumor core. HistoMap successfully bridges bulk RNA-seq and spatial single-cell architectures. Our findings provide a molecular rationale for immune checkpoint blockade resistance and identify the SPP1-fibroblast axis as a pivotal target for therapeutic sensitization.

## 1. Introduction

Spatial diversity and cellular heterogeneity are fundamental hallmarks of tissue complexity [1]. Recent advances in spatially resolved transcriptomics have enabled the characterization of cellular composition, molecular architecture, and functional nuances at unprecedented spatial scales. Contemporary state-of-the-art experimental technologies primarily comprise imaging-based and sequencing-based approaches, which involve trade-offs between high-throughput cell detection, unbiased mRNA discovery, and single-cell resolution [2,3]. While there is a growing demand for spatially resolved single-cell transcriptomic profiling across clinical and research cohorts to investigate molecular variance in biological and pathological processes with heightened granularity, current methodologies remain hampered by protracted experimental timelines, prohibitive costs, and limited scalability [1,4].

Over the past two decades, extensive investment in RNA sequencing (RNA-seq) has led to its pervasive application in transcriptomic analysis, catalyzing landmark initiatives such as the Encyclopedia of DNA Elements (ENCODE) [5], The Cancer Genome Atlas (TCGA) [6], and the International Cancer Genome Consortium (ICGC) [7]. Repurposing these vast repositories—particularly the extensive bulk RNA-seq datasets—to unveil cellular diversity and spatial expression patterns represents a formidable yet profoundly significant endeavor.

Current computational tools, such as CPM, CIBERSORT, and MuSiC, are restricted to inferring cell-type proportions from bulk RNA-seq data; they lack the capacity to reconstruct single-cell profiles or map them onto physical tissue coordinates [8,9,10,11,12]. In parallel with classical deconvolution methods, generative deep learning frameworks have emerged as a powerful paradigm for synthetic single-cell data generation. Generative Adversarial Networks (GANs) operate through an adversarial interplay between a generator, which synthesizes synthetic samples, and a discriminator, which attempts to distinguish them from empirical observations. This process continues until the generator produces outputs effectively indistinguishable from genuine data. Conditional GANs (cGANs) further refine this paradigm by integrating auxiliary information as conditioning variables, thereby facilitating controlled and targeted data generation. While these architectures have demonstrated significant utility in single-cell RNA-seq imputation and augmentation—effectively modeling complex cell-state landscapes—they remain prone to inherent limitations. Specifically, challenges such as mode collapse and training instability can severely impede their capacity to fully capture the vast, continuous spectrum of biological heterogeneity, highlighting a critical need for more robust generative methodologies. Consequently, the development of a framework capable of effectively deconvolving massive bulk RNA-seq data into spatially resolved single-cell expression profiles holds the potential to simultaneously elucidate cellular heterogeneity and spatial transcriptomic shifts within complex tissue architectures.

Here, we introduce HistoMap, a deep learning-based spatial deconvolution framework designed to reconstruct spatial expression profiles at single-cell resolution by integrating histology–transcriptomic data across diverse tissue samples. Leveraging high-quality single-cell RNA sequencing (scRNA-seq) [13], spatial transcriptomics (ST) [14] references, and H&E-stained histology images, HistoMap transforms bulk transcriptomic data into spatially resolved single-cell expression maps. We posit that the bulk transcriptome can be conceptualized as a weighted ensemble of single-cell expression profiles within a defined cellular manifold, analogous to the stochastic sampling and barcoding of individual cells from a tissue mass. HistoMap first synthesizes single-cell transcriptomic data within this manifold to identify a cellular subset whose aggregate profile aligns with the bulk observations. Subsequently, by synergizing ST reference information with image-derived spatial topological constraints, the model employs dual guidance—gene expression and morphology—to precisely map generated single cells to their optimal spatial coordinates. This facilitates high-resolution cell-type localization within their native tissue architecture. Collectively, HistoMap demonstrates robust performance across varied datasets and experimental conditions, elucidating cellular diversity and spatial expression heterogeneity in complex tissues while broadening the application of deep learning in the biomedical sciences.

## 2. Results

### 2.1. Design Concept of HistoMap

To address the inherent limitations of bulk transcriptomics in capturing spatial heterogeneity, we present HistoMap—an integrated deep learning framework for reconstructing high-resolution, spatially resolved single-cell expression profiles. While bulk measurements provide comprehensive transcriptomic coverage, they lack the cellular and spatial granularity required to decipher complex tumor microenvironments. HistoMap bridges this gap by synergistically integrating single-cell RNA-seq (scRNA-seq) references, spatial transcriptomics (ST), and histological images.

**Figure 1:**
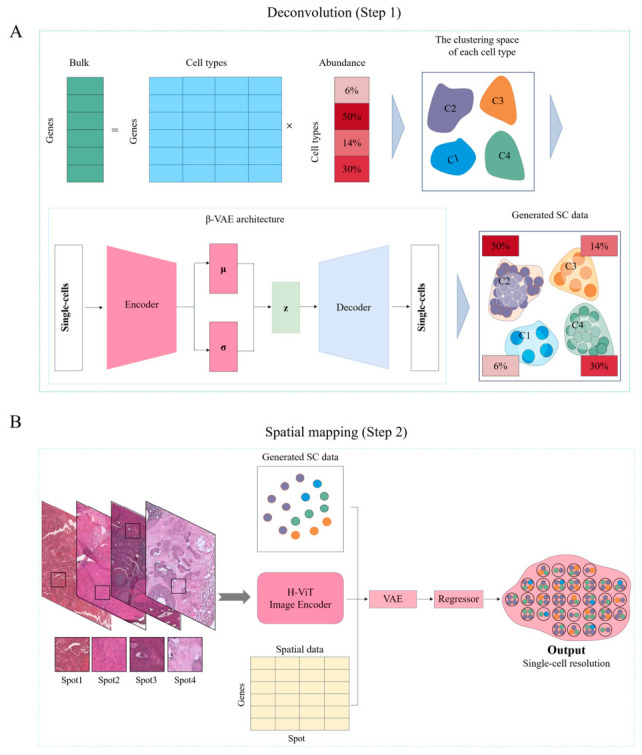
Overview of the HistoMap framework for single-cell spatial deconvolution of bulk transcriptomes: (**A**) Deconvolution of bulk transcriptome data; (**B**) Image-guided spatial mapping.

The overall architecture of the HistoMap methodology is summarized in Figure 1. The HistoMap methodology is implemented as a two-stage computational pipeline. The initial stage leverages a β-variational autoencoder (β-VAE) model [15], utilizing scRNA-seq as a reference to deconvolve bulk transcriptomic data into high-fidelity single-cell expression profiles. The subsequent spatial mapping stage employs an H-ViT (Histological Vision Transformer) architecture to encode morphological features from H&E-stained images [16,17,18]. By integrating these morphological embeddings with spatial transcriptomic references and image-derived topological adjacency constraints, the model precisely localizes the synthesized single cells to their most probable spatial coordinates through the synergistic optimization of gene expression and histological morphology.

### 2.2. Performance Evaluation of the Deconvolution Step of HistoMap

**Figure 2:**
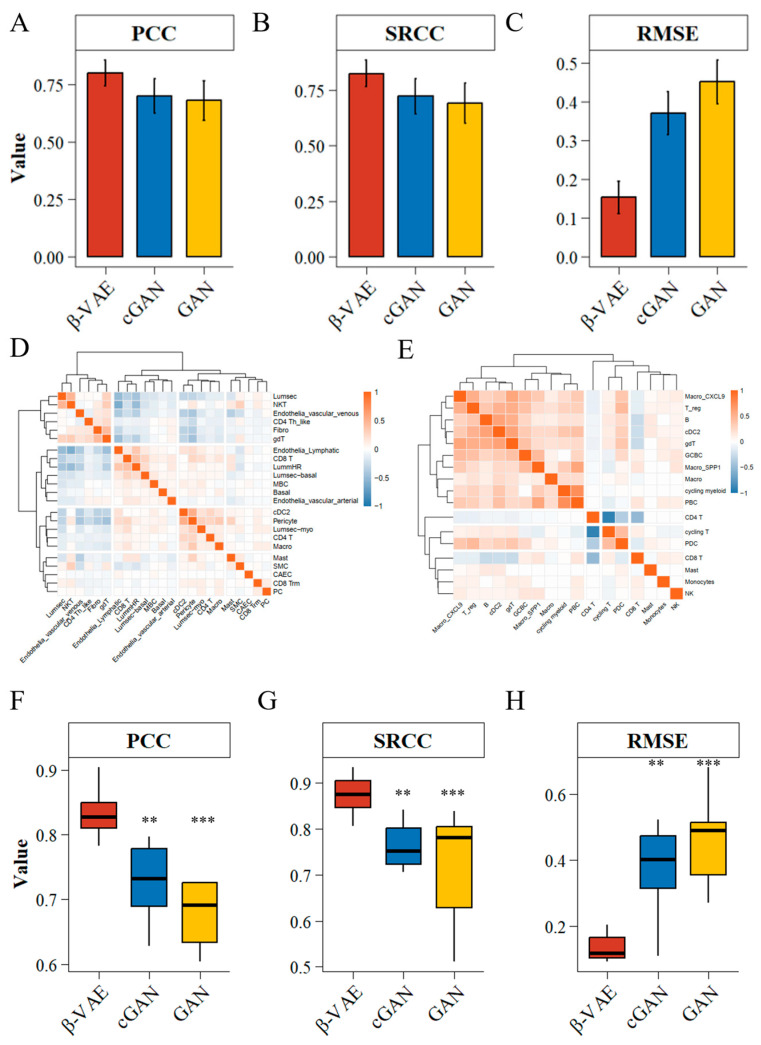
Comprehensive benchmarking of HistoMap deconvolution performance across multi-organ transcriptomic datasets: (**A**–**C**) Evaluation of deconvolution accuracy using a standardized 8:1:1 dataset split. Performance was quantified across multiple tissue types using (**A**) Pearson Correlation Coefficient (PCC) to assess linear expression fidelity, (**B**) Spearman’s Rank Correlation Coefficient (SRCC) to evaluate monotonic preservation of gene rankings, and (**C**) Root Mean Square Error (RMSE) to measure absolute quantification deviance. (**D**,**E**) Heatmaps illustrate the high consistency between marker gene expression in HistoMap-synthesized data and ground-truth single-cell profiles for both (**D**) breast cancer and (**E**) colorectal cancer. The diagonal alignment (or high correlation) confirms that HistoMap successfully recaptures cell-type-specific molecular signatures during the deconvolution process. (**F**–**H**) Assessment of model stability via ten-fold cross-validation. Consistent distributions of (**F**) PCC, (**G**) SRCC, and (**H**) RMSE. Asterisks above cGAN and GAN boxes indicate statistically significant differences from the β-VAE model, assessed by pairwise Wilcoxon signed-rank tests following a global Friedman test (** *p* < 0.01, *** *p* < 0.001).

To evaluate the robustness of the HistoMap deconvolution module, we performed benchmarking using 50 unpaired simulations—where bulk and single-cell data were derived from the same patient but obtained from independent datasets. These simulations encompassed eight high-quality scRNA-seq datasets spanning diverse human tissues, including the brain, breast, liver, lung, intestine, tonsil, prostate, and skin (Detailed specifications of the datasets used in this study are available in Table S1). Since traditional deconvolution methods such as CPM [10], CIBERSORT [9], and ImmuCC [19] are limited to predicting cell-type proportions rather than gene expression profiles, we compared HistoMap with Generative Adversarial Networks (GANs) [20] and Conditional GANs (CGANs) [21]. As illustrated in Figure 2A–C, HistoMap achieved significantly higher Pearson correlation coefficients and lower Root Mean Square Error (RMSE) for gene expression compared to the other two generative frameworks. While CGAN demonstrated performance comparable to HistoMap in single-cell synthesis, its training stability and inference robustness were markedly lower. A detailed comparison of the correlation between cell-type-specific marker gene expression in synthesized data and ground-truth single-cell types is presented in Figure 2D,E.

To further investigate the robustness of HistoMap single-cell generation, we assessed the predictive stability of the models using ten-fold cross-validation. Samples from the eight tissue types were randomly partitioned into ten folds, each consisting of a training set (90%) and a validation set (10%), ensuring no overlap between validation sets across folds. HistoMap demonstrated consistent robustness and effectively mitigated overfitting (Figure 2F–H).

### 2.3. Performance Evaluation of the Spatial Mapping Step of HistoMap

**Figure 3:**
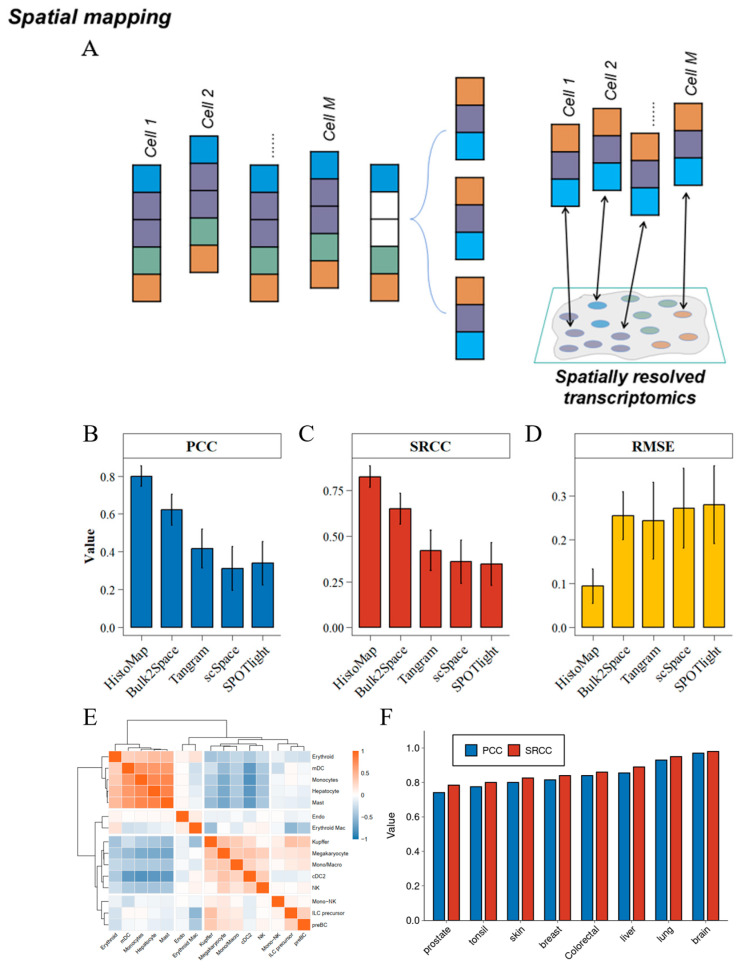
Benchmarking the spatial mapping performance of HistoMap across multi-organ transcriptomic datasets: (**A**) Schematic of the spatial mapping phase. HistoMap anchors generated single cells to precise tissue coordinates using spatially resolved transcriptomic references. (**B**–**D**) Quantitative evaluation of mapping accuracy via an 8:1:1 data-splitting strategy. Performance metrics were calculated to assess the concordance between predicted and observed spatial distributions, including (**B**) PCC for linear expression similarity, (**C**) SRCC for preservation of relative gene expression rankings, and (**D**) RMSE for absolute quantification deviance. (**E**) Validation of cell-type-specific signatures in liver cancer. (**F**) Cross-tissue performance benchmarking and generalizability assessment.

In this stage, HistoMap assigns generated single cells to spatial coordinates based on spatially resolved transcriptomic references (Figure 3A). We utilize spatial barcoding-based RNA-seq technologies, including Spatial Transcriptomics (ST) [14,22], High-Definition Spatial Transcriptomics (HDST) [23], Slide-seq [24,25], and 10× Genomics Visium [26], etc. These methods are typically constrained by tissue micro-regions (spots) of fixed geometries and fail to achieve true single-cell resolution. To evaluate mapping accuracy, we conducted a comprehensive comparison using 200 paired simulation datasets, benchmarking HistoMap against four established methods: Tangram [27], scSpace [28], SPOTlight [29], and Bulk2Space [30]. These methods serve as benchmarks for optimizing cell-type composition and spatial gene expression.

**Figure 4:**
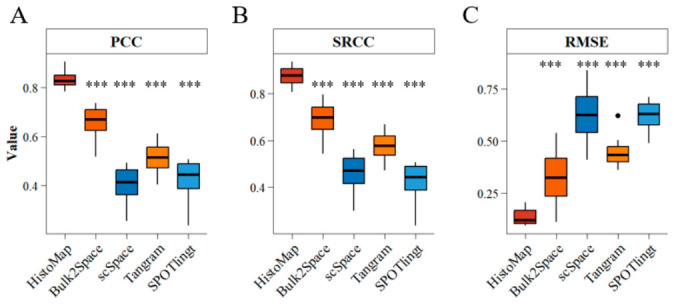
Systematic evaluation of model predictive stability via ten-fold cross-validation across diverse tissues: (**A**–**C**) Multi-dimensional benchmarking of spatial mapping performance. Results are derived from ten-fold cross-validation across eight human tissue types, comparing HistoMap with baseline methodologies. Metrics include (**A**) PCC, (**B**) SRCC, and (**C**) RMSE. Asterisks above the Bulk2Space, scSpace, Tangram, and SPOTlight boxes indicate statistically significant differences from the HistoMap model (*** *p* < 0.001).

As shown in Figure 3B–D, HistoMap achieved the highest gene expression correlation (PCC = 0.800) and a significantly lower RMSE of around 0.153 compared to its competitors. Furthermore, the cell-type compositions and spatial distributions predicted by HistoMap exhibit high concordance with the ground-truth values (Figure 3E). Figure 3F illustrates HistoMap’s performance across eight tissue types; using PCC and SRCC as evaluation metrics, HistoMap demonstrates consistent performance across all datasets, underscoring its robust spatial mapping capabilities across diverse organ systems. To further assess predictive stability, we implemented a ten-fold cross-validation framework. Samples from the eight tissue types were randomly partitioned into ten folds, each comprising a training set (90%) and a non-overlapping validation set (10%). The predictive performance across models is detailed in Figure 4. Regarding PCC (Figure 4A), HistoMap yielded both the highest predictive accuracy of 0.832 and superior stability. The value of the standard deviation is 0.036, outperforming all other models. While Bulk2Space and Tangram showed relatively high accuracy and stability, scSpace and SPOTlight exhibited poor performance in both metrics. This suggests that scSpace and SPOTlight are less suitable for prediction tasks involving significant inter-individual variation between single-cell and spatial transcriptomic samples. Consistent trends were observed for SRCC and RMSE (Figure 4B,C), with HistoMap consistently achieving the best scores across all indicators, further consolidating its definitive advantage in single-cell spatial mapping tasks.

To further validate the robustness of HistoMap, we performed ten-fold cross-validation using a non-paired scRNA-seq reference (Figure S1). The results demonstrate that HistoMap maintains stable performance across different data partitions, does not rely on specific sample pairings, and shows no evidence of overfitting to particular patients. This further supports the generalizability of HistoMap to unseen samples from the same organ.

### 2.4. Ablation Study

We conducted ablation experiments to explore the impact of histology–transcriptomic integration—specifically the integration of gene expression profiles and histological images—on the predictive performance of HistoMap. To investigate whether the synergistic utilization of transcriptomic and morphological information as initial input features can enhance the model’s performance, we conducted two variant studies, denoted as OnlyGene and OnlyIma, which utilize expression data and image-derived features in isolation, respectively.

**Figure 5:**
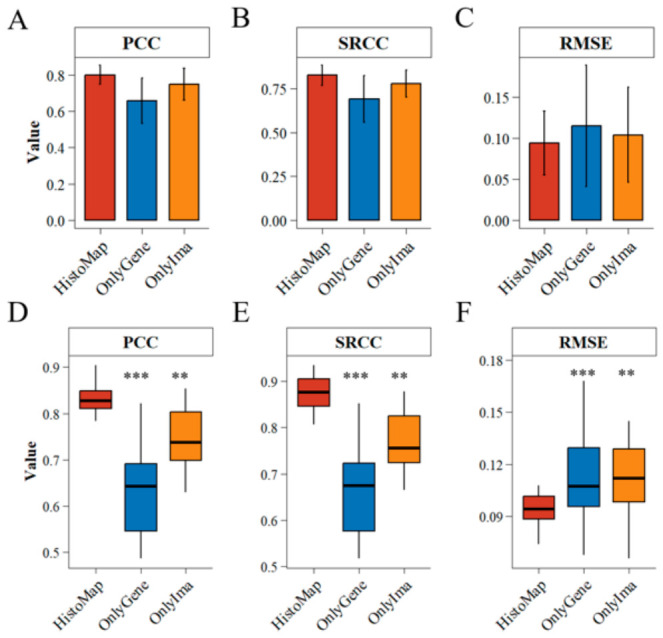
Ablation studies characterizing the necessity of multi-modal feature fusion: (**A**–**C**) Cross-tissue performance benchmarking. Evaluation of HistoMap against unimodal variants (OnlyGene and OnlyIma) across diverse organ systems using (**A**) PCC, (**B**) SRCC, and (**C**) RMSE; (**D**–**F**) Stability assessment via ten-fold cross-validation. Boxplots illustrate the distribution of (**D**) PCC, (**E**) SRCC, and (**F**) RMSE across validation folds. Asterisks above the OnlyGene and OnlyIma boxes indicate statistically significant differences from the HistoMap model (** *p* < 0.01, *** *p* < 0.001).

Figure 5A–C present the results of the two ablation experiments across multiple tissue types, while Figure 5D–F provides a comprehensive comparison between HistoMap and its ablation variants. From Figure 5A–C, it can be observed that utilizing either gene expression data (OnlyGene) or histological images (OnlyIma) in isolation resulted in significantly lower performance, with PCC values of 0.658 and 0.747, respectively, compared to the full HistoMap model.

Figure 5D–F demonstrates a similar trend across ten-fold cross-validation. While HistoMap consistently achieved the highest mean values and the lowest standard deviations for PCC, SRCC, and RMSE, the ablation variants exhibited greater fluctuations and diminished stability under varying conditions. Therefore, we can infer that the integration of multimodal omics data—specifically the synergy between transcriptomic profiles and morphological topologies—significantly improves the predictive accuracy and robustness of the model by capturing higher-dimensional spatial architectures.

Notably, the superior performance of OnlyIma relative to OnlyGene suggests that H&E-stained images provide a higher information density for spatial mapping tasks. Consequently, when specific transcriptomic data is unavailable or corrupted, the image-based feature extraction module can still provide relatively accurate predictions, serving as a reliable alternative input to maintain the model’s practical utility in data-constrained scenarios.

### 2.5. Case Study: Spatial Reconstruction and Evaluation of the Macro_SPP1-Mediated Immune-Excluded Microenvironment in Colon Cancer

**Figure 6:**
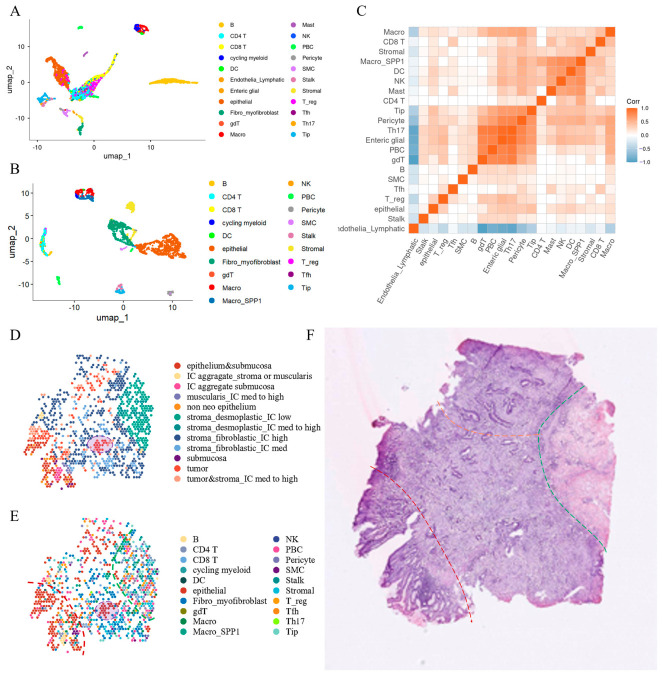
High-precision single-cell spatial reconstruction and histological validation in colorectal cancer: (**A**,**B**) Benchmarking synthesized single-cell profiles against reference datasets. UMAP visualizations illustrate the clustering topology of (**A**) the reference single-cell dataset and (**B**) HistoMap-generated “pseudo-single cells”; (**C**) Transcriptomic fidelity of the reconstructed atlas. (**D**,**E**) Validation of spatial mapping accuracy against pathological gold standards. (**D**) Expert-curated pathological annotations defining distinct histological functional zones including tumor nests, epithelium, and desmoplastic stroma. (**E**) Spatial distribution of individual generated cells, where HistoMap localizes reconstructed single-cell profiles to their predicted tissue coordinates. In (**D**), the purple circles denote tumor regions; in (**E**), these same circles are occupied by epithelial cells. (**F**) Representative H&E-stained histological section.

We conducted a case study to evaluate the accuracy of our model’s single-cell mapping by reconstructing the spatial architecture of colon cancer patients. Single-cell spatial reconstruction analysis was performed on 14 cases, with the resulting clustering space of the reference dataset and the algorithm-generated cells shown in Figure 6A,B. In the clustering space, the generated “pseudo-single cells” exhibited cell subtype abundances highly consistent with the reference data and demonstrated excellent overlap in UMAP space. Furthermore, Figure 6C demonstrates that the expression of the top 30 highly specific marker genes for each cell subtype (detailed in Supplementary Table S2) in the HistoMap-generated atlas is highly correlated with the reference data, confirming the biological accuracy of our spatial transcriptomic reconstruction. Collectively, these results confirm the high biological validity and transcriptomic fidelity of the single-cell atlas generated via deconvolution.

Subsequently, each cell was mapped to its precise spatial coordinates based on the spatial reference (Figure 6D). To correlate spatial molecular expression patterns with histological features, we examined the molecular composition of annotated regions within the spatial reference (Figure 6E). To evaluate the accuracy of the HistoMap algorithm, the predicted cell type distributions were benchmarked against gold-standard annotations provided by expert pathologists. The spatial alignment revealed exceptional consistency between molecularly defined cell populations and histological functional zones. Specifically, predicted epithelial and tumor cell clusters were accurately localized within the “Tumor” and “Epithelium & Submucosa” regions, validating the algorithm’s capability to identify malignant domains. Furthermore, stromal lineages—particularly myofibroblasts (Fibro_myofibroblast)—were strictly confined within the desmoplastic stroma, with spatial boundaries aligning precisely with the histological structures of the submucosa and muscularis. This robust spatial alignment underscores the reliability of our mapping method in preserving the spatial coordinates of cell identities.

**Figure 7:**
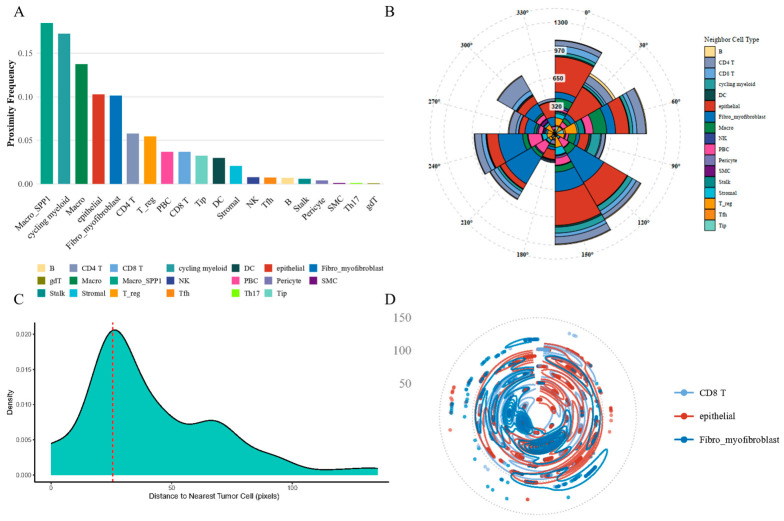
Macro_SPP1-mediated spatial sequestration and the formation of an immune-excluded desmoplastic barrier: (**A**) Neighborhood enrichment analysis of Macro_SPP1 social preferences; (**B**) Spatial rose plots illustrating neighborhood anisotropy. Angular sectors (30° bins, 0–330°) show orientation relative to the cell center. The radial axis represents cumulative stacked cell counts for 17 neighboring cell types. Dashed concentric circles denote abundance scale intervals; (**C**) Quantitative spatial distance analysis relative to the tumor boundary; (**D**) Radial density distribution analysis of Macro_SPP1.

The integrated spatial atlas revealed a complex tumor microenvironment (TME) architecture characterized by a prominent desmoplastic response. We observed a non-random peripheral distribution of SPP1+ macrophages (Macro_SPP1), which were specifically enriched at the tumor-stroma interface (invasive front). To investigate the spatial interaction characteristics of Macro_SPP1, we assessed its social preferences through neighborhood enrichment analysis (Figure 7A) [31,32,33]. The results demonstrated highly specific spatial co-localization, with Macro_SPP1 exhibiting significant interaction frequencies with myofibroblasts and other myeloid cells. Utilizing spatial rose plots to analyze the anisotropy of its neighborhood (Figure 7B), we found that the stromal components surrounding Macro_SPP1 displayed a polarized, directional distribution. This topological arrangement supports the role of Macro_SPP1 as a “spatial hub” that collaborates with fibroblasts to construct a pro-fibrotic niche, forming a continuous cellular “barrier” around malignant nests [31,34,35]. Additionally, the localized enrichment of angiogenesis-related endothelial cells (Tip and Stalk cells) within these stromal channels highlights the spatial synergy between neoangiogenesis and fibroblast activation [36]. These findings suggest that the desmoplastic stroma acts not only as a structural scaffold but also as a functional niche regulating tumor progression.

Analysis of the immune topology identified a prominent “immune-excluded” phenotype within the samples. Despite the presence of substantial CD4+ T, CD8+ T, and B cell populations, they were largely restricted to the distal stroma or immune cell aggregates (IC aggregates) within the submucosa, as indicated by pathological annotations. Quantitative spatial distance analysis (Figure 7C) confirmed that Macro_SPP1 was significantly enriched within a narrow zone approximately 50–100 pixels from the tumor boundary, forming a physical “sequestration belt.” Radial density distribution analysis (Figure 7D) revealed a precipitous decline in the density of cytotoxic CD8+ T cells within regions highly occupied by Macro_SPP1 and its associated fibroblasts. This spatial exclusion indicates that the barrier composed of Macro_SPP1 and fibroblasts serves as a functional obstruction to T cell recruitment into the tumor core. Furthermore, the co-existence of regulatory T cells (Tregs) in these peripheral regions further exacerbates the local immunosuppressive state [36,37,38].

Based on these spatial mapping findings, we propose a molecular rationale for targeted therapeutic strategies in colon cancer. The Macro_SPP1-mediated desmoplastic barrier induces spatial T-cell exclusion, suggesting that conventional immune checkpoint blockade (ICB) monotherapy may exhibit diminished efficacy in colon cancer due to restricted physical accessibility. This is consistent with established literature, which characterizes MSS/pMMR and immune-excluded colorectal cancer as largely refractory to ICB monotherapy [39]. Consequently, clinical interventions targeting SPP1-mediated signaling or TGF-β-driven stromal remodeling may be required to disrupt this physical and chemical barrier, thereby “sensitizing” the TME and promoting immune cell infiltration. Moreover, the enrichment of angiogenic Tip cells in specific stromal niches supports the potential of combining anti-angiogenic agents with immunotherapy to improve drug delivery to the tumor core through vessel normalization.

## 3. Discussion

By integrating the single-cell resolution and transcriptomic coverage of scRNA-seq with the spatial positioning capabilities of spatial transcriptomics, we propose HistoMap, a novel single-cell spatial deconvolution framework. Our model merges spatial transcriptomic references with image-derived topological constraints to precisely map reconstructed single cells to their optimal tissue coordinates. Under the dual guidance of gene expression and morphology, HistoMap achieves high-resolution cell-type localization within a spatial context, effectively overcoming the inherent limitations associated with various clinical sample types. Extensive experiments demonstrate that our model delivers superior performance in single-cell spatial mapping tasks across diverse experimental conditions. Furthermore, we conducted external validation to confirm the robustness and generalizability of our method across different data batches.

A case study employing high-precision single-cell spatial reconstruction systematically reveals the topological characteristics of the tumor invasive front in colorectal cancer, confirming that Macro_SPP1 (SPP1+ macrophages) serves as a central orchestrator driving the spatial remodeling of the tumor microenvironment (TME). This cell subpopulation, through anisotropic high-density aggregation with myofibroblasts at the tumor-stroma interface, constructs a continuous desmoplastic barrier. This spatial architecture not only physically restricts the infiltration of cytotoxic T cells into the tumor core but also functionally defines an “immune-excluded” microenvironment, acting as a critical spatial determinant of tumor immune evasion.

We observed distinct differences in cell-type representation between the reference scRNA-seq atlas and the HistoMap-generated outputs. Mast cells appeared in the reference but not in our reconstruction, while enteric glial cells (EGCs) were uniquely resolved by HistoMap. These discrepancies reflect the interplay between data quality and model optimization in our β-VAE framework. The β-VAE’s optimization—prioritizing robust transcriptional features—acts as a denoising filter, likely suppressing sparse signals such as those from mast cells. Conversely, the emergence of EGCs demonstrates the framework’s ability to extract subtle biological signals from bulk transcriptomics. By integrating image-derived topological constraints with global transcriptomic information, HistoMap recovers fragile or underrepresented populations often missed by standard scRNA-seq. This differential capture underscores the model’s fidelity to high-confidence spatial mapping and reveals its potential to identify unannotated niches, enhancing resolution of the spatial tumor microenvironment.

A fundamental conceptual distinction separates HistoMap from traditional non-spatial deconvolution algorithms like CIBERSORT. While traditional methods quantify cell-type abundances strictly within a single bulk sample, HistoMap integrates multimodal inputs to project single-cell transcriptional profiles onto an independent histological matrix. Because the reference single cells and the target tissue section lack a shared physical origin, the resulting map represents a probabilistic spatial realization rather than a deterministic recording of a specific sample’s exact ground truth. Nevertheless, this reconstruction is strictly bounded by conserved organ-level architectures and tissue morphology. By capturing generalizable spatial priors—such as the recurrent co-localization of immune cells and tumor parenchyma—HistoMap effectively translates bulk signals into a highly plausible histological context. This probabilistic framework provides a robust, generalizable strategy to decipher complex tissue architectures, such as the immune-excluded microenvironment, when matched spatial transcriptomics data are unavailable.

Ultimately, HistoMap will assist clinicians in accurately quantifying cell–cell interactions and predicting the efficacy of immunotherapy, offering new perspectives and tools for deciphering complex biological processes and advancing the clinical translation of precision medicine. To assist clinicians and researchers in deciphering the tumor microenvironment, the complete source code and tutorial for HistoMap are fully open-source and available on GitHub at: https://github.com/stat-hj/HistoMap.git (accessed on 2 June 2026). In future work, we aim to further refine the feature extraction module to more efficiently capture composite structural features, better utilize multi-modal information, and continue enhancing the model’s predictive power.

## 4. Materials and Methods

### 4.1. Datasets

All scRNA-seq, spatial transcriptomics (ST), and bulk RNA-seq datasets employed in this study were retrieved from high-quality peer-reviewed publications, the Gene Expression Omnibus (GEO) [40], and TCGA [6]. To ensure data integrity, unannotated, ambiguous, or low-quality cells were excluded from the analysis. Comprehensive metadata for each dataset are summarized in Table S1. The study cohort comprises a total of 558 samples spanning eight human organ systems, each featuring integrated ST data, matched scRNA-seq references, and corresponding H&E-stained histology images. The total dataset was randomly partitioned into training, validation, and testing sets at a ratio of 8:1:1. Specifically, the training set was utilized for HistoMap parameter optimization, while the validation set was employed in conjunction with an early stopping strategy to facilitate model selection and prevent overfitting. The testing set served as an internal benchmark to evaluate the model’s performance in reconstructing single-cell spatial architectures. The specific tissue and organ types included in the cohort are detailed in Table S3.

### 4.2. Data Processing

Transcriptomic data preprocessing was performed using R (version 4.4.0). For the scRNA-seq datasets, 2000 cells with ambiguous annotations were excluded, and cell types represented by fewer than 10 cells were removed to ensure statistical robustness. All single-cell analyses were conducted following the standard workflow of the Seurat package (version 5.4.0) [41]. To mitigate technical noise and ensure high data quality, stringent quality control (QC) filtering was applied: genes detected in fewer than three cells and cells with fewer than 200 detected genes (indicative of low-quality or empty droplets) were filtered out. The remaining data were processed using the “LogNormalize” [41] method to account for variations in sequencing depth. For ST data, empty barcodes and spots containing “NA” expression values were removed, followed by standard analysis within the Seurat framework. To harmonize the scRNA-seq and ST data across different experimental platforms, the ComBat algorithm was employed for batch effect correction [42,43], aligning both modalities onto a consistent numerical scale. Regarding the H&E histology images, preprocessing was implemented to eliminate technical biases arising from batch-to-batch staining variations and physical tissue deformations. Stain normalization was applied to correct for color inconsistencies, while image registration [44] was utilized to align multiple tissue sections into a unified spatial coordinate system. These steps established a reliable foundation for subsequent cross-section spatial integration and multi-modal analysis.

### 4.3. The HistoMap Framework

#### 4.3.1. Stage 1: Deconvolution of Bulk Transcriptome Data

**Cell-Type Proportion Prediction.** For a given bulk RNA-seq dataset, we first estimate the constituent cell-type proportions, which subsequently guide the generation of spatially resolved single-cell gene expression profiles. A schematic overview of the bulk transcriptome deconvolution pipeline is presented in Figure S2. Since the cell-type identities and corresponding expression signatures can be derived from the scRNA-seq reference, we aggregated cells of the same type and calculated their mean expression profiles. The average vector $c_{i}\in {\mathbb{R}}^{N}$ is defined as the characteristic gene expression profile of a specific cell type i ($i\in \left\{1,2,\ldots ,C\right\}$), where C represents the total number of cell types. Given the gene expression vector $x\in {\mathbb{R}}^{N}$ of a bulk RNA-seq dataset, the cell-type proportions are modeled as follows:

(1) $$\sum_{i=1}^{C}p_{i}c_{i}=x$$

Here, $p_{i}$ is the proportions vector for cell type i.

Subsequently, Least Squares Estimation (LSE) [11,45] is applied to estimate $p_{i}$ by minimizing the squared discrepancies between the observed bulk data and the predicted mixture:

(2) $$\underset{p}{\min}{\left\|x-Cp\right\|}_{2}$$

In this formulation, $C\in {\mathbb{R}}^{N\times C}$ represents the cell type gene expression profile, where each column corresponds to a different cell type, and $p={\left[p_{1},p_{2},\ldots ,p_{C}\right]}^{T}$ is the vector of proportions to be estimated.

**Single-cell simulation by β-VAE**. Let $D=\left\{X,V,W\right\}$ represent a dataset where gene expression vectors $x\in {\mathbb{R}}^{N}$ are governed by two distinct sets of generative factors: conditionally independent factors $v\in {\mathbb{R}}^{K}$, where $\log p(v|x)=\sum_{k}\log p(v_{k}|x)$, and conditionally dependent factors $w\in {\mathbb{R}}^{H}$. We assume x is synthesized by a ground-truth simulator S such that $p_{\theta }\left(x|v,w\right)=S\left(v,w\right)$, where θ is the generative model parameter. Our objective is to learn an unsupervised generative model $p_{\theta }\left(x|z\right)$ that approximates this simulator: $p_{\theta }\left(x|z\right)\approx p_{\theta }\left(x|v,w\right)=S\left(v,w\right)$. Given that the marginal likelihood $p_{\theta }\left(x\right)$ and true posterior $p_{\theta }\left(z|x\right)$ are intractable, we introduce a variational distribution $q_{\phi }\left(z|x\right)$ to approximate the posterior and capture v in a disentangled manner. In this latent space, individual units are sensitive to specific generative factors while remaining invariant to others, facilitating robust knowledge generalization. Meanwhile, the dependent factors w may remain entangled within a latent subspace of z orthogonal to v.

To minimize the KL divergence between the approximate and true posteriors, we define the variational lower bound $L\left(\theta ,\phi ;x\right)$ as:

(3) $$L\left(\theta ,\phi ;x\right)=E_{q_{\phi }\left(z|x\right)}\left[\log\left(p_{\theta }\left(x|z\right)\right)\right]-D_{\mathrm{KL}}\left(q_{\phi }\left(z|x\right)∥p\left(z\right)\right)$$

To promote representation disentanglement, we treat the latent space as an information bottleneck by constraining $q_{\phi }\left(z|x\right)$ to align with an isotropic Gaussian prior $p\left(z\right)∼N\left(0,I\right)$. This constrained optimization is expressed as:

(4) $$\underset{\phi ,\theta }{\max}E_{q_{\phi }\left(z|x\right)}\left[\log p_{\theta }\left(x|z\right)\right]\;\;\;s.t.\;\;\;D_{\mathrm{KL}}\left(q_{\phi }\left(z|x\right)∥p_{\theta }\left(z\right)\right)<\varepsilon$$

where ε denotes the bottleneck capacity. Applying the Karush-Kuhn-Tucker (KKT) conditions, we formulate the Lagrangian:

(5) $$F\left(\theta ,\phi ,\beta ;x,z\right)=E_{q_{\phi }\left(z|x\right)}\left[\log p_{\theta }\left(x|z\right)\right]-\beta \left(D_{\mathrm{KL}}\left(q_{\phi }\left(z|x\right)∥p_{\theta }\left(z\right)\right)-\varepsilon \right)$$

Here, the multiplier β serves as a regularization coefficient that penalizes latent channel capacity. Under complementary slackness, this yields the *β*-VAE objective:

(6) $$L_{\beta -\mathrm{VAE}}\left(\theta ,\phi ;x\right)=E_{q_{\phi }\left(z|x\right)}\left[\log p_{\theta }\left(x|z\right)\right]-\beta \cdot D_{\mathrm{KL}}\left(q_{\phi }\left(z|x\right)∥p\left(z\right)\right)$$

We propose that setting β>1 imposes a more stringent bottleneck, forcing the model to discover the most informative, decoupled generative factors. While a high β necessitates a trade-off between reconstruction fidelity and disentanglement quality, an optimal balance allows disentangled representations to emerge naturally through compressed latent encoding.

#### 4.3.2. Stage 2: Mapping Reconstructed Single Cells to Tissue Coordinates

**Image Preprocessing and Feature Extraction via Vision Transformer.** Given an input image I with dimensions H×W×C (where H, W, and C denote height, width, and channel count, respectively), we employ a sliding window strategy of fixed size to partition the image into a regularly arranged set of local patches. The patch size is defined as P×P, with a sliding stride s (typically $s\in \left\{112,224\right\}$) to modulate the degree of overlap between adjacent patches.

The resulting set of image patches is then fed into a Vision Transformer (ViT) model [16]. We utilize a pre-trained ViT as a static feature extractor, denoted as the encoder: $f_{\mathrm{ViT}}\left(\cdot ;\theta \right):{\mathbb{R}}^{224\times 224\times 3}$, with model parameters θ. Throughout all subsequent experiments, these encoder parameters remain frozen:

(7) $$\theta \equiv \theta_{0},\;\nabla_{\theta }L=0$$

which implies that gradients are not computed and no parameter updates are performed. Features are extracted via this fixed encoder as:

(8) $$z_{i}=f_{\mathrm{ViT}}\left({\mathrm{Patch}}_{i};\theta_{0}\right)\in {\mathbb{R}}^{768},\;\forall i$$

Finally, the extracted feature vectors $\left\{z_{i}\right\}$ undergo L2 normalization:

(9) $${\tilde{z}}_{i}=\frac{z_{i}}{{\left\|z_{i}\right\|}_{2}}\in {\mathbb{R}}^{768}$$

where ${\left\|z_{i}\right\|}_{2}=\sqrt{\sum_{k=1}^{768}z_{i,k}^{2}}$ represents the Euclidean norm (L2 norm) of the vector. The resulting normalized feature vectors $\left\{{\tilde{z}}_{i}\right\}$ are subsequently utilized for spatial coordinate prediction.

**Spatial Mapping.** In this framework, the VAE structure was systematically simplified by transitioning it into a deterministic Autoencoder (AE) [46] dedicated to feature representation learning. The current Encoder architecture is defined as:

(10) $$N_{x}\to 1024\to 512\to 256=N_{z}$$

where $N_{x}$ represents the input feature dimensionality. This configuration utilizes a three-layer fully connected (dense) hidden structure with progressively decreasing dimensions to compress the input $N_{x}$ into a low-dimensional latent representation $N_{z}$. In this deterministic setup, the Encoder directly outputs the latent feature vector:

(11) $$z=\mathrm{Encoder}\left(x\right),\;z\in {\mathbb{R}}^{256}$$

Correspondingly, the Decoder is implemented as a single-layer linear mapping:

(12) $$\tilde{x}=Wz+b,\;W\in {\mathbb{R}}^{3000\times 256}$$

where W denotes the weight matrix and $\tilde{x}$ represents the reconstructed output.

The training objective for this model omits the Kullback–Leibler (KL) divergence term, focusing solely on the reconstruction loss:

(13) $$\underset{\theta }{\min}E_{x∼D}\left[{\left\|x-Decoder(Encoder(x))\right\|}_{2}^{2}\right]$$

where θ represents the model parameters and ${\left\|\cdot \right\|}_{2}^{2}$ denotes the squared L2 norm, equivalent to the Mean Squared Error (MSE). Following the completion of training, the Encoder is utilized as a static feature extractor. Its generated latent representations z are subsequently fed into two independent Random Forest Regression models [47] to predict the x and y spatial coordinates, respectively (detailed workflow shown in Figure 1B).

#### 4.3.3. Evaluation Metrics for Predictive Performance

To systematically assess the spatial mapping performance of the proposed model, this study established an evaluation framework across two dimensions: correlation and error.

Correlation Metrics: The Pearson Correlation Coefficient (PCC) and Spearman’s Rank Correlation Coefficient (SRCC) were utilized to quantify the consistency between the reconstructed single-cell spatial structures and the reference spatial transcriptomics. Both PCC and SRCC range from −1 to 1; values approaching 1 indicate a higher degree of correlation between the predicted spatial coordinates and the ground truth. PCC and SRCC were employed to provide complementary assessments of spatial mapping performance. While PCC quantifies linear relationships and is sensitive to the magnitude of continuous variables, SRCC evaluates monotonic relationships based on rank order, offering robustness against non-normal distributions and potential outliers. The dual application of these metrics ensures a comprehensive evaluation of prediction accuracy, capturing both linear consistency and rank-order preservation.

Error Metrics: The Root Mean Square Error (RMSE) was adopted to measure the deviation between the predicted single-cell spatial distribution and the reference spatial transcriptomic distribution. Given that RMSE is highly sensitive to prediction errors, lower values represent superior localization precision and a more robust capacity for distribution fitting.

#### 4.3.4. Neighborhood Enrichment Analysis

To evaluate the spatial interaction preferences of the Macro_SPP1 population, we performed a neighborhood enrichment analysis based on the spatial coordinates reconstructed by HistoMap. For each Macro_SPP1 cell, we defined its spatial neighborhood using a k-nearest neighbor (k-NN) search constrained within a radius of 150 units. The top 30 nearest neighbors within this radius (excluding the query cell itself) were retained to characterize the local cellular microenvironment.

The composition of each neighborhood was quantified as the proportional distribution of surrounding cell types. To assess whether the enrichment of specific cell types (e.g., myofibroblasts) around Macro_SPP1 was statistically significant, we implemented a permutation-based approach. Specifically, we randomly shuffled cell-type labels across all spatial coordinates 1000 times to generate a null distribution. An enrichment score was then calculated by comparing the observed neighborhood composition to the mean of the null distribution, with statistical significance defined as *p* < 0.05.

## Data Availability

No primary data were generated in this study. All data analyzed are derived from publicly available repositories, with detailed source information, including database names and accession numbers, provided in Supplementary Table S1. The source code and implementation of the HistoMap framework are publicly accessible via GitHub: https://github.com/stat-hj/HistoMap.git (accessed on 2 June 2026).

## Supplementary Materials

The following supporting information can be downloaded at: https://www.mdpi.com/article/10.3390/ijms27125259/s1.

## Abbreviations

The following abbreviations are used in this manuscript:

Bulk RNA-seq	Bulk RNA sequencing
ENCODE	Encyclopedia of DNA Elements
H&E	Hematoxylin and eosin stains
H-ViT	Histopathology Vision Transformer
ICGC	International Cancer Genome Consortium
PCC	Pearson Correlation Coefficient
RMSE	Root Mean Square Error
SRCC	Spearman’s Rank Correlation Coefficient
ST	Spatial Transcriptomics
scRNA-seq	Single-cell RNA sequencing
TCGA	The Cancer Genome Atlas
TME	Tumor Microenvironment
β-VAE	β-Variational Autoencoder
