# Supplementary material for: HistoMap: Reconstructing Spatially Resolved Single-Cell Profiles from Bulk RNA-Seq to Decipher the Immune-Excluded Microenvironment in Colon Cancer
Source: Int J Mol Sci. 2026 Jun 10;27(12):5259. doi: 10.3390/ijms27125259 (PMC13300051; doi:10.3390/ijms27125259)
Supplement: Supplementary file 1 [file ijms-27-05259-s001.zip › ijms-4309879-supplementary-Table S1.pdf]

Table S1. Summary of data sources

URL	Tissue
https://zenodo.org/records/7961605	Breast
https://zenodo.org/records/10373041	Tonsil
https://data.mendeley.com/datasets/svw96g68dv	Prostate
https://5locationslung.cellgeni.sanger.ac.uk/	Lung
https://www.ncbi.nlm.nih.gov/geo/query/acc.cgi?acc=GSE242311	Breast
https://www.ncbi.nlm.nih.gov/geo/query/acc.cgi?acc=GSE243367	liver
https://www.ncbi.nlm.nih.gov/geo/query/acc.cgi?acc=GSE232766	Brain
https://www.ncbi.nlm.nih.gov/geo/query/acc.cgi?acc=GSE213688	Breast
https://www.ncbi.nlm.nih.gov/geo/query/acc.cgi?acc=GSE226663	Brain
https://www.ncbi.nlm.nih.gov/geo/query/acc.cgi?acc=GSE243291	Tonsil
https://www.ncbi.nlm.nih.gov/geo/query/acc.cgi?acc=GSE243179	Breast
https://www.ncbi.nlm.nih.gov/geo/query/acc.cgi?acc=GSE249729	Skin
https://www.ncbi.nlm.nih.gov/geo/query/acc.cgi?acc=GSE226533	Skin
https://www.ncbi.nlm.nih.gov/geo/query/acc.cgi?acc=GSE243981	liver
https://www.ncbi.nlm.nih.gov/geo/query/acc.cgi?acc=GSE245388	Prostate
https://www.ncbi.nlm.nih.gov/geo/query/acc.cgi?acc=GSE254364	liver
https://www.ncbi.nlm.nih.gov/geo/query/acc.cgi?acc=GSE218951	Breast
https://www.ncbi.nlm.nih.gov/geo/query/acc.cgi?acc=GSE237183	Brain
https://explore.data.humancellatlas.org/projects/2fe3c60b-ac1a-4c61-9b59-f6556c0fce63	Lung
https://spatial-skin-atlas.cellgeni.sanger.ac.uk/	Skin
https://www.ncbi.nlm.nih.gov/geo/query/acc.cgi?acc=GSE235672	Brain
https://www.ncbi.nlm.nih.gov/geo/query/acc.cgi?acc=GSE225857	Intestine
https://www.ncbi.nlm.nih.gov/geo/query/acc.cgi?acc=GSE215897	Lung

Continued Table S1. Summary of data sources

URL	Tissue
https://ngdc.cncb.ac.cn/crost/download	liver
https://ngdc.cncb.ac.cn/crost/download	Brain
https://ngdc.cncb.ac.cn/crost/download	Prostate
https://www.ncbi.nlm.nih.gov/geo/query/acc.cgi?acc=GSE206391	Skin
https://soar.fsm.northwestern.edu/#databrowser	Brain
https://soar.fsm.northwestern.edu/#databrowser	Skin
https://soar.fsm.northwestern.edu/#databrowser	Skin
https://doi.org/10.1002/path.5857	Prostate
https://soar.fsm.northwestern.edu/#databrowser	Brain
https://doi.org/10.1158/0008-5472.CAN-22-2682	Breast
http://lifeome.net/supp/livercancer-st/data.htm	liver
https://cellxgene.cziscience.com/collections/4195ab4c-20bd-4cd3-8b3d-65601277e731	Breast
https://www.ncbi.nlm.nih.gov/geo/query/acc.cgi?acc=GSE206552	liver
https://spatial.rhesusbase.com/datasets/dataPage/STW-H-Skin-Visium-1	Skin
https://spatial.rhesusbase.com/datasets/dataPage/STW-H-Liver-Visium-5	liver
https://spatial.rhesusbase.com/datasets/dataPage/STW-H-Liver-Visium-4	liver
https://spatial.rhesusbase.com/datasets/dataPage/STW-H-Liver-Visium-3	liver
https://spatial.rhesusbase.com/datasets/dataPage/STW-H-Liver-Visium-3	liver
https://spatial.rhesusbase.com/datasets/dataPage/STW-H-Liver-Visium-2	liver
https://spatial.rhesusbase.com/datasets/dataPage/STW-H-Liver-Visium-1	liver
https://spatial.rhesusbase.com/datasets/dataPage/STW-H-Kidney-Visium-1	liver
https://www.10xgenomics.com/resources/datasets/human-Breast-cancer-block-a-section-1-1-standard-1-1-0	Breast
https://www.10xgenomics.com/resources/datasets/human-Breast-cancer-block-a-section-2-1-	Breast

Continued Table S1. Summary of data sources

URL	Tissue
https://www.10xgenomics.com/resources/datasets/human-Breast-cancer-whole-transcriptome-analysis-1-standard-1-2-0	Breast
https://www.10xgenomics.com/resources/datasets/human-colorectal-cancer-whole-transcriptome-analysis-1-standard-1-2-0	Intestine
https://www.10xgenomics.com/resources/datasets/human-glioblastoma-whole-transcriptome-analysis-1-standard-1-2-0	Brain
https://www.10xgenomics.com/resources/datasets/human-Breast-cancer-ductal-carcinoma-in-situ-invasive-carcinoma-ffpe-1-standard-1-3-0	Breast
https://www.10xgenomics.com/resources/datasets/human-prostate-cancer-adenocarcinoma-with-invasive-carcinoma-ffpe-1-standard-1-3-0	Prostate
https://www.10xgenomics.com/resources/datasets/normal-human-prostate-ffpe-1-standard-1-3-0	Prostate
https://www.10xgenomics.com/resources/datasets/human-prostate-cancer-acinar-cell-carcinoma-ffpe-1-standard	Prostate
https://www.10xgenomics.com/resources/datasets/human-Breast-cancer-visium-fresh-frozen-whole-transcriptome-1-standard	Breast
https://www.10xgenomics.com/resources/datasets/human-lung-cancer-ffpe-2-standard	Lung
https://www.10xgenomics.com/resources/datasets/human-colorectal-cancer-11-mm-capture-area-ffpe-2-standard	Intestine
https://www.10xgenomics.com/resources/datasets/human-brain-cancer-11-mm-capture-area-ffpe-2-standard	Brain
