# Supplementary material for: HistoMap: Reconstructing Spatially Resolved Single-Cell Profiles from Bulk RNA-Seq to Decipher the Immune-Excluded Microenvironment in Colon Cancer
Source: Int J Mol Sci. 2026 Jun 10;27(12):5259. doi: 10.3390/ijms27125259 (PMC13300051; doi:10.3390/ijms27125259)
Supplement: Supplementary file 1 [file ijms-27-05259-s001.zip › ijms-4309879-supplementary-Table S3.pdf]

Table S3. Composition and distribution of the included tissue samples (partial)

Sample Name	Tissue	Split
Pt13	Brain	Testing Set
Pt18	Brain	Testing Set
Pt19	Brain	Testing Set
Pt24	Brain	Testing Set
Pt3	Brain	Testing Set
p1_biopsy2_rna	Brain	Testing Set
CA1	Breast	Testing Set
CA7	Breast	Testing Set
LN2	Breast	Testing Set
LN8	Breast	Testing Set
P03	Breast	Testing Set
CCL_s0813	Colon	Testing Set
CCL_s1231	Colon	Testing Set
SMC01-N	Colon	Testing Set
SMC23-T	Colon	Testing Set
SMC24-T	Colon	Testing Set
SMC25-T	Colon	Testing Set
4834STDY7038750	Liver	Testing Set
C70_TST	Liver	Testing Set
C72_TST	Liver	Testing Set
CISE06	Liver	Testing Set
CCL_s0813	Liver	Testing Set

Continued Table S3. Composition and distribution of the included tissue samples (partial)

Sample Name	Tissue	Split
P5_Normal	Skin	Testing Set
P6_Normal	Skin	Testing Set
P9_Normal	Skin	Testing Set
bcc_ear2	Skin	Testing Set
BCLL10-1	Tonsil	Testing Set
BCLL12-3	Tonsil	Testing Set
BCLL13-1	Tonsil	Testing Set
BCLL13-2	Tonsil	Testing Set
BCLL26-1	Tonsil	Testing Set
BCLL6-2	Tonsil	Testing Set
BCLL9-2	Tonsil	Testing Set
patient1-2	Tonsil	Testing Set
patient1-5	Tonsil	Testing Set
Pt10	Brain	Training Set
Pt11	Brain	Training Set
Pt12	Brain	Training Set
Pt14	Brain	Training Set
Pt15	Brain	Training Set
Pt16	Brain	Training Set
Pt2	Brain	Training Set
Pt20	Brain	Training Set
CA3	Breast	Training Set
CA4	Breast	Training Set
CA5	Breast	Training Set
