# Supplementary figures and images for: HistoMap: Reconstructing Spatially Resolved Single-Cell Profiles from Bulk RNA-Seq to Decipher the Immune-Excluded Microenvironment in Colon Cancer
Source: Int J Mol Sci. 2026 Jun 10;27(12):5259. doi: 10.3390/ijms27125259 (PMC13300051; doi:10.3390/ijms27125259)

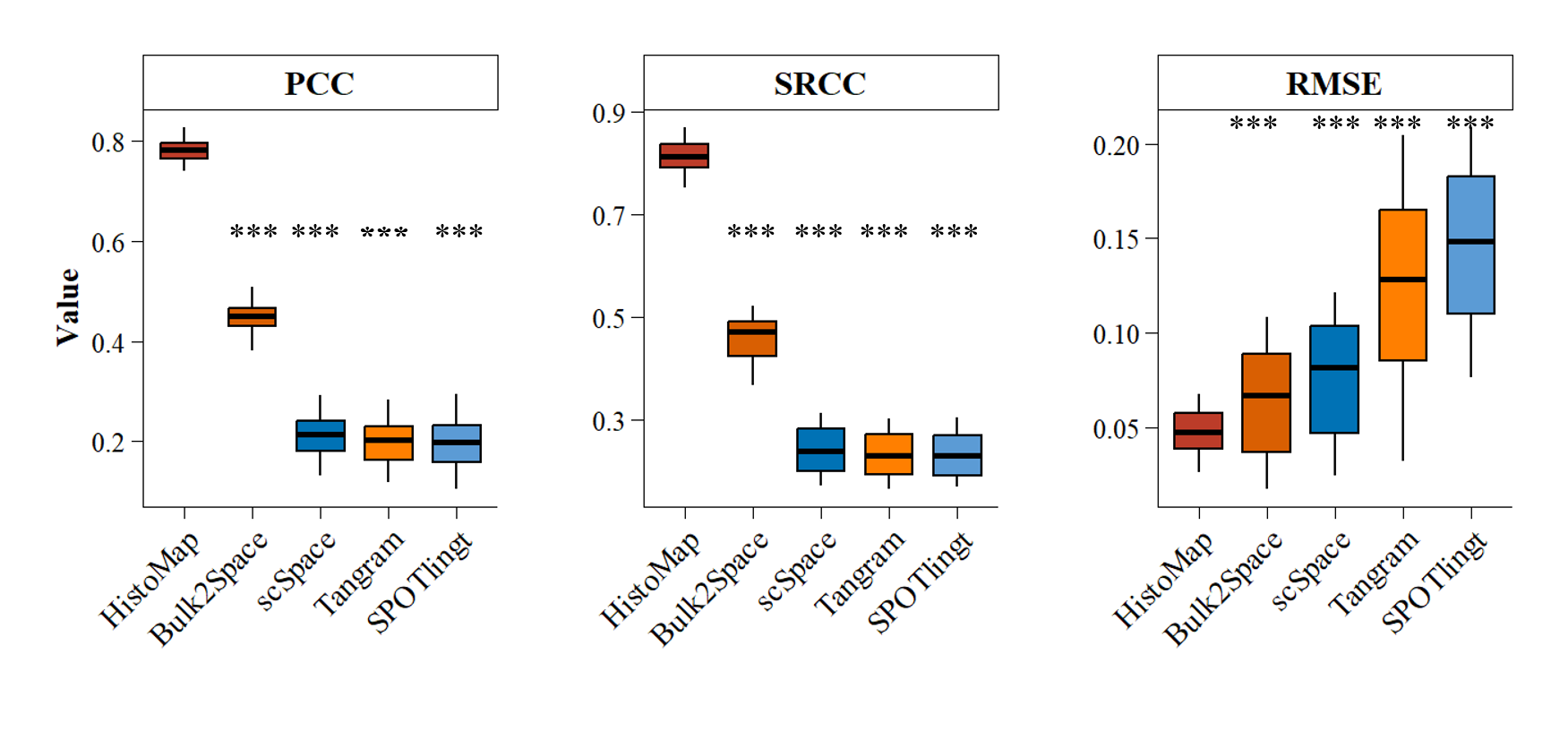

Supplement: Supplementary file 1 [file ijms-27-05259-s001.zip › ijms-4309879-supplementary-Figure S1.tif]

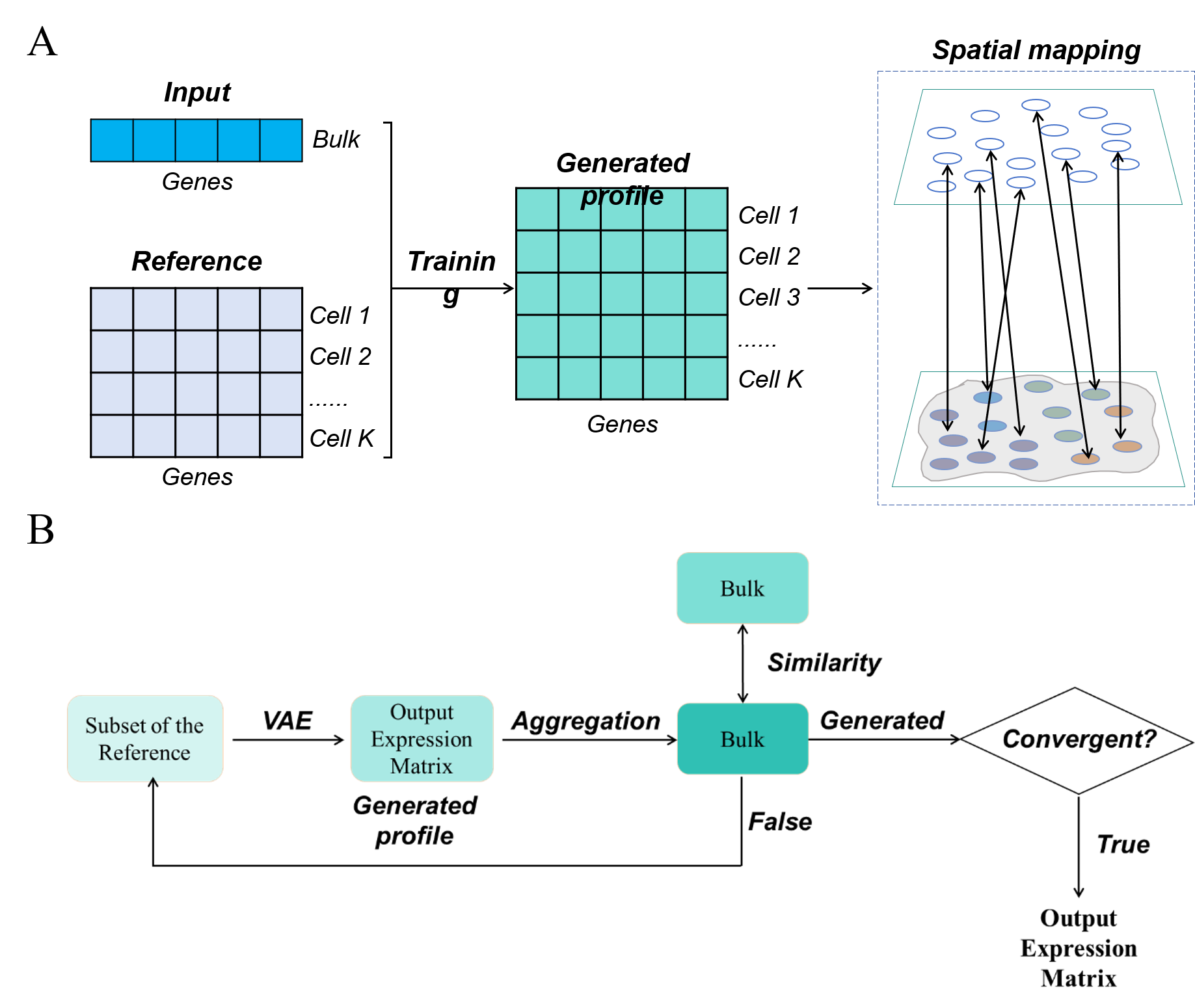

Supplement: Supplementary file 1 [file ijms-27-05259-s001.zip › ijms-4309879-supplementary-Figure S2.tif]
